# The transcription of *MGAT4A *glycosyl transferase is increased in white cells of peripheral blood of Type 2 Diabetes patients

**DOI:** 10.1186/1471-2156-8-73

**Published:** 2007-10-22

**Authors:** Eduardo López-Orduña, Miguel Cruz, Jaime García-Mena

**Affiliations:** 1 Departamento de Genética y Biología Molecular, Centro de Investigación y de Estudios Avanzados. Av IPN 2508, Col Zacatenco Mexico DF 07360, Mexico; 2 Unidad de Investigación Médica en Bioquímica, Centro Médico Nacional Siglo XXI, Instituto Mexicano del Seguro Social, Mexico City, Mexico

## Abstract

**Background:**

Human glycosylase IV is involved in GLUT2 transporter regulation in pancreatic β cells. A KO of this gene along with a high fat diet in a mice model has been associated with the development of type 2 diabetes (T2D). The aims of this study were to measure and compare the *MGAT4A *mRNA levels in white blood cells (WBC) from T2D subjects and healthy subjects (T2NB), and to measure the half-life of the *MGAT4A *mRNA.

**Results:**

We studied a sample of 73 individuals, 40 T2D subjects and 33 T2NB subjects. Anthropometrical and biochemical profiles were registered. The *MGAT4A *mRNA levels in WBC and the transcript half-life in Jurkat T cells were determined by Real-Time PCR. A blood differential cell counting was made for each individual. Cell counting showed T2D subjects exhibited an increased number of WBC compared to T2NB subjects (*P *= 0.0001). Biochemical parameters such as fasting glucose (*P *= 0.0001), and triglycerides (*P *= 0.002) were statistically significant. T2D subjects had 4.2-fold more *MGAT4A *transcript compared to T2NB subjects (*P *= 0.002). The *MGAT4A *mRNA had a half-life of 2.04 h in Jurkat T cells.

**Conclusion:**

The results of this work suggest that in T2D subjects, high levels of glucose and triglycerides are accompanied by an increase on *MGAT4A *mRNA levels and WBC count; condition that suggests a pro-inflammatory state due to a chronic metabolic stress.

## Background

The human *MGAT4A *gene encodes a glycosyltransferase regulating the formation of tri- and multiantennary branching structures on membrane proteins that require glycosylation as signal for its exportation in the Golgi apparatus [1]. This gene is located in the chromosome 2 (2q12), and the 8382 bases mRNA encoding the isozyme A (NCBI, NM_012214) contains 15 introns, 16 exons and encodes a protein of 535-aminoacids (NCBI, NP_036346). The *MGAT4A *gene is well conserved as demonstrate the 96% identity on the primary aminoacid sequence shared between the *MGAT4A *and the bovine GnT-IV glycosilases [1]. Transcripts of 9.7, 7.6, 5.1, 3.8, and 2.4 kb have been detected by Northern-blot in several human tissues and human derived cell lines; and the relative expression levels of these transcripts were similar among the different tissues and cell lines tested, with higher expression in spleen, thymus, peripheral blood leukocytes, lymph node, prostate, pancreas, and small intestine [1]. In the case of the cell lines tested, the promyelocytic leukemia cell line HL-60 and the lymphoblastic leukemia cell line MOLT-4 showed the highest expression [1]. The human glycosylase IV is a type II membrane protein, similar to other known Golgi glycosyltransferases [2]. *MGAT4A *has three known isoforms and may play a role regulating the availability of serum glycoproteins, oncogenesis, and cell differentiation [1,3].

Oligosaccharide antigens are commonly used as tumor markers and many studies have shown these antigens can modulate tumor cell adhesion, motility, and invasiveness [3]. Recently the *MGAT4A *was identified as a genetic marker for pancreatic cancer in addition to its known role in choriocarcinoma development [4]. It is remarkable since reports of defects in protein glycosylation are becoming increasingly associated with a range of human diseases [2].

It has been shown that high counts of peripheral white blood cells (WBC) are associated with insulin resistance and type 2 diabetes (T2D) [5-7]. Concomitant to this increment, polymorphonuclear and mononuclear cells can be activated by advanced glycation end products [8], oxidative stress [9,10], and cytokines in a state of hyperglycemia, typical features of T2D [11]. On the other hand lymphocytes have acquired significance like cell model to study the expression of several biomarkers, since they offer a convenient alternative to invasive methods of diagnosis aimed to measure the expression of several important T2D associated genes [12,13].

The important role of *MGAT4A *function in the development of T2D was shown in studies using engineered mice lacking *MgaT4a*. These mice were phenotypically hyperglycemic, hypoinsulinemic and had impaired glucose tolerance [14]. The role of *MgaT4a *on controlling the traffic of GLUT2 in pancreatic β cells was described in these mice, linking a high fat diet with the development of experimental diabetes in mice [14].

As has been mentioned before, alterations in the expression of this gene have been implicated in the development of several human diseases such as choriocarcinoma and pancreatic cancer [3,4], showing that defective function of this gene is of pathological consequence for the organism. We hypothesized *MGAT4A *gene expression might be affected in individuals suffering of T2D. With this purpose we determined the level of MGAT4A expression in white cells of peripheral blood of well characterized T2D patients and healthy subjects, and measure the half-life of the transcript in a model lymphocyte T cell line.

## Results

### T2D phenotype biochemical tests and WBC counts

The clinical and biochemical profile of the subjects under study are summarized in the Table 1. T2D subjects have an average of seven years of evolution, and the average age of the selected group of subjects in both categories are comparable, guaranteeing the healthy condition of controls. In the T2D group, the biochemical values for glucose, HDL and LDL parameters are in concordance with the phenotype of the disease. The BMI for both groups indicates overweight, what is a common feature in nowadays Mexico City's inhabitants. The diastolic and systolic blood pressures in both groups show absence of hypertension and none of the participants had cardiovascular complications at the time of the study. Neither group of subjects received anti-hypertensive drugs to control the blood pressure. The blood cell count showed T2D exhibit an increase number of WBC compared to the T2NB group (*P *= 0.0001) (Table 1).

**Table 1:** Clinical characteristics of the participants in the study

	T2D	T2NB	*P*
n (male/female)	40 (20/20)	33 (17/16)	
Age (years)	51.26 ± 7.77	51.19 ± 4.70	0.96
BMI (Kg/m^2^)	27.87 ± 3.80	26.80 ± 3.04	0.18
SBP (mm Hg)	124.61 ± 16.02	118.50 ± 8.27	0.05
DBP (mm Hg)	79.58 ± 10.29	76.09 ± 6.55	0.09
Glucose (mmol/l)	9.71 ± 4.04	4.91 ± 0.36	0.0001
Cholesterol (mmol/l)	5.64 ± 1.23	5.47 ± 0.88	0.50
HDL (mmol/l)	1.17 ± 0.26	1.24 ± 0.41	0.37
LDL (mmol/l)	3.60 ± 0.88	3.43 ± 1.54	0.55
Triglycerides (mmol/l)	2.54 ± 1.49	1.67 ± 0.61	0.002

WBC (cells/ml)	8.19 × 10^6 ^± 6.64 × 10^2^	5.90 × 10^6 ^± 8.59 × 10^2^	0.0001

Data are means ± SD; T2D, Type 2 Diabetes subjects; T2NB, healthy no T2D background unrelated subjects.

### MGAT4A mRNA expression levels

**Figure 1:**
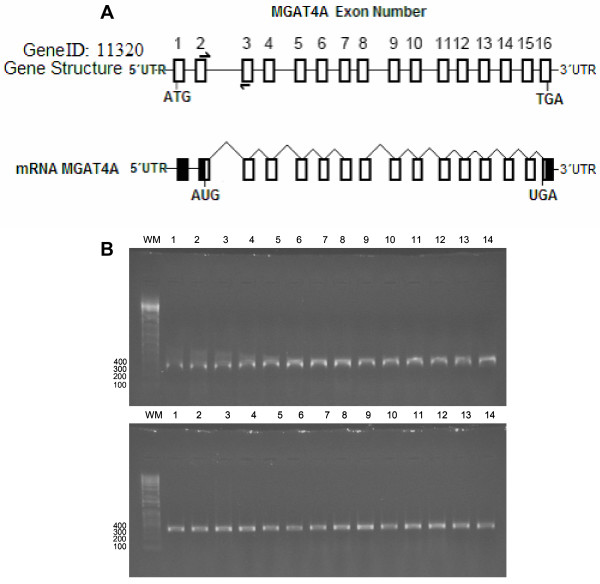
A) The gene structure for MGAT4A gene. The MGAT4A exons are shown as rectangles in the DNA and mRNA molecules; introns are indicated as a continuous line in DNA and break lines in the mRNA molecule. Primers GNTF and GNTR amplify a 323 bp continuous portion of exons 2 and 3. Figure not to scale. Gene information, NCBI, NM_012214. B) 1% agarose gel fractionation of the 323 bp PCR product amplified from cDNA made from healthy subjects (top) and T2D subjects (bottom). WM: 100 bp molecular weight marker.

**Figure 2:**
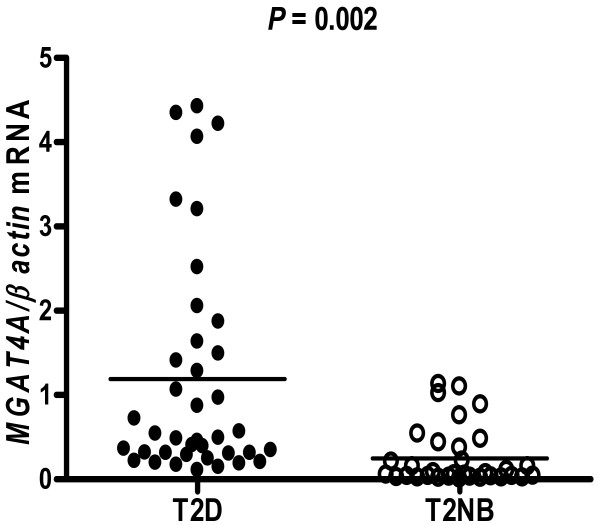
MGAT4A mRNA expression in the phenotypic groups. Individuals in each phenotypic category were grouped according to the clinic and biochemical parameters as T2D, type 2 Diabetes subjects and T2NB, healthy no T2D background subjects. mRNA levels were quantified by Real-Time PCR and normalized to mRNA levels of β-actin. Results are expressed as the means ± SE. ANOVA P values are shown.

We detected the presence on *MGAT4A *transcripts in WBC, employing Real-Time PCR and primers designed to amplify a 323 bp region the of *MGAT4A *transcript (Figure 1B). The *MGAT4A *mRNA expression levels quantified in WBC (Figure 2) revealed a 4.2-fold increase in mRNA levels in T2D subjects with respect to T2NB subjects (T2D subjects 1.19 ± 0.21 vs. T2NB subjects, 0.25 ± 0.06, *P *= 0.002).

### MGAT4A mRNA half-life determination

**Figure 3:**
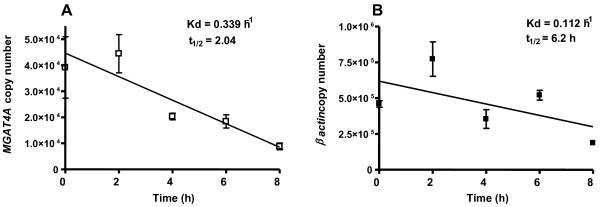
mRNA half life determination. Cells were treated with 1 μM of Actinomycin D to block mRNA synthesis. mRNA decay was measured by detection of the 323 bp PCR product in Jurkat cells A) β-actin mRNA decay was measured by detection of 587 bp in the same cells B) The values represent the mean ± SE of (empty squares) or β-actin (filled squares) copy number per μg of total RNA from three independent experiments made by duplicate.

The *MGAT4A *mRNA half life was determined using Jurkat lymphocyte T-cell line as a model under Actinomycin D general RNA synthesis inhibition. The Figure 3 shows a half-life of 2.04 h for *MGAT4A *as measured by copy number detection in Jurkat T cells (Kd = 0.339 h^-1^). The β-actin mRNA half-life in the same cells was of 6.17 h (Kd = 0.112 h^-1^) as measured by detection of the 597 pb β-actin fragment.

## Discussion

In this work we measured the *MGAT4A *mRNA transcript concentration present in WBC from T2D subjects and healthy no T2D background unrelated subjects, as well as measured the half-life on this transcript in the Jurkat T cell line used as model. Our results shows that *MGAT4A *gene expression in WBC from T2D patients exhibited higher levels of transcripts concentration compared to the healthy subjects (Figure 2). This result is remarkable since in the mice model, the *MGAT4A *transcripts levels have been reported low, while the animals are phenotipically hyperglycemic, hypoinsulinemic and had impaired glucose tolerance [14,15]. One explanation to this result is we measured the transcripts in WBC and not in pancreatic β-cells being this difference due to differential tissue specific gene expression. However a more interesting plausible explanation is the higher level of *MGAT4A *gene expression exhibited by the T2D subjects, is a consequence of a low pro-inflammatory state derived of chronic hyperglycaemia, a common condition in T2D patients [16-18]. Interestingly the WBC count was higher in the T2D subjects compared to the healthy individuals, suggesting that the increase in *MGAT4A *transcripts levels and WBC count are related with the metabolic disorder observed in T2D patients. In the development of T2D it is emerging the knowledge of the existence of many genes that are down or up-regulated during the development of the disease as is demonstrated in animal models [12].

The *MGAT4A *transcript exhibits a half-life of 2.04 h, measured by Real-Time PCR in the human Jurkat T cell line used like model in this work (Figure 3); this half-life classifies this mRNA in the so called protein modification group of transcripts, with an average half-life of <5 h [19].

Thus we propose that an increase in MGAT4A transcripts level and augmented WBC number in the bloodstream contribute to a pro-inflammatory state and other metabolic alterations observed in T2D patients. This explanation is based on the observation that high levels of glucose and triglycerides contributes to high stress environment for the WBC, mainly lymphocytes [20]. This stress could trigger the activation of this type of cells, increasing even more the WBC count and the *MGAT4A *transcript level, but only in subjects with a particular T2D genetic background. We hypothesize this condition does not occurs in healthy people prior to the onset of any clinical manifestation of the disease or prior to the hyperinsulinemia present in obese individuals or T2D patients with recent diagnosis.

## Conclusion

We report a high expression of MGAT4A mRNA in WBC of T2D patients; and we consider that glycosylase IVA involved in WBC proliferation and activation associated with the pro-inflammatory condition found in most cases of T2D patients, is one of the several gene products with an important role in the pathogenesis events triggering diabetes. We also show white blood cells provide a sample useful to measure the expression of these genes, which permits to acquire data to infer about the pro-inflammatory condition of the individuals in relation with diabetes. We also found that the *MGAT4A *mRNA belongs to the protein modification group of transcripts, with an average half-life of less than 5 h.

## Methods

### Subjects Profile

A population-based register of individuals belonging to low and medium economic ranks living in Mexico City were selected from a T2D cohort. The case-control sample consisted of 73 individuals divided in two phenotypic categories: 40 patients diagnosed with T2D within the last 7 years and 33 healthy unrelated subjects without T2D background obtained from the Blood Bank at Centro Medico Siglo XXI, IMSS in Mexico City. Diagnosis of T2D was established according to the American Diabetes Association criteria. The study protocol was approved by the IMSS Ethics Committee and informed consent was obtained from all participants, in accordance with the Helsinki Declaration revised in 2000. Patients with active infections within the last two months or surgery within the last three months previous to the test; with affections as cancer; T1D, cardiopathy; or with any pharmacological treatment (with exception of T2D) were excluded from the study.

### Biochemical studies

Participants were scheduled for clinical laboratory evaluation following a 12 hour overnight fasting, and were required to collect urine in the 24 h prior to the clinic visit. Blood samples were taken to assess levels of fasting glucose, total cholesterol, triglycerides and high and low density cholesterol levels (HDL, LDL). All the participants were interviewed by a physician, and weight and height were measured to calculate body mass index (BMI = kg/m^2^). Systolic and diastolic blood pressure measurements were made using a mercurial sphygmomanometer (Hitachi) from both arms and the average of two measurements was obtained. Biochemical measurements were assayed for all subjects using a ILab 350 Clinical chemistry System (Instrumentation laboratory IL). LDL-cholesterol concentrations were derived from lipid values according to the Friedewald formula [21].

### Cell culture

The Jurkat T cell line was cultured in 75 ml bottles with 20 ml of RPMI-1640 medium (Sigma) supplemented with 2 mM glutamine, 1.5 g/L NaHCO_3_, glucose 25 mM, 0.1 mg/ml gentamicin and 10% heat inactivated Bovine Calf Serum. Fresh medium was added according to standard tissue culture techniques. Viability was verified with Trypan Blue (Sigma).

### White blood cells count

The total red blood cell count; total white blood cell count and the fraction for platelets was made using an automated blood cell counter (Sysmex K-4500, TOA Medicals Electronics), using a 4 ml sample of peripheral blood from each individual.

### RNA extraction and RT-PCR

Total RNA was purified directly from 1.0 × 10^7 ^WBC and from 2.2 × 10^6 ^Jurkat cells using a QIAamp RNA kit (Qiagen) following the manufacturer's instructions. Integrity of DNase I treated RNA was verified by electrophoresis in 1.0% agarose gel stained with ethidium bromide and its concentration was measured by spectrophotometry at 260/280 nm. cDNA was synthesized using 1 μg of total RNA by RT-PCR with Super Script-II Reverse transcription enzyme (Invitrogen) and random primers (Invitrogen) in a GeneAmp PCR System 2700 (Applied Biosystems).

### Cloning of MGAT4A and β-actin gene fragments

A 323 bp region of the *MGAT4A *gene (NCBI, NM_012214) was amplified from cDNA (Figure 1A) using the primers GNTF 5'-TTG GCC TAG AGC CAG GAG TA-3' and GNTR 5'-ACA CGC TTG AAC TGT TGC AC-3', and a 597 bp region of the β-actin gene (NCBI, NM_001101) was amplified from cDNA using the primers BAF 5'-CCA AGG CCA ACC GCG AGA AGA TGA C-3' and BAR 5'-AGG GTA CAT GGT GGT GCC GCC AGA C-3'. PCR products were cloned into pCR-Blunt II TOPO (Invitrogen) to generate plasmids pTOPO-GNT323 and pTOPO-BA597, respectively. The plasmids were purified using the midi plasmid kit (Qiagen) according to the manufacturer's protocol and quantified by 260/280 nm spectrophotometry. Cloned fragments were sequenced using Big Dye terminator reagent v3.1 (Applied Biosystems) using primers Sec-For 5'-CAG GAA ACA GCT ATG A-3' and Sec-Rev 5'-CTG GCC GTC GTT TTA-3'. Analysis of sequencing reactions was made in an ABI Prism 310 (Applied Biosystems).

### Quantitation of MGAT4A mRNA using Real-Time PCR

Detection of the *MGAT4A *was performed by Real-Time-PCR using primers GNTF and GNTR to amplify a 323 bp product and BAF and BAR primers to amplify a 597 bp β-actin product. Serial ten-fold dilutions ranging from 10^4 ^to 10^8 ^molecules for each plasmid were prepared. Each standard curve was constructed with averaged data of three independent measurements made by triplicate. These curves were used to estimate the initial transcript copy number for *MGAT4A *and β-actin in cDNA samples prepared from total RNA. Real-Time PCR reactions were performed in a 10 μl of reaction volume using 1/10 of the 20 μl cDNA reaction as initial template. The DNA Fast Start SYBR-green Kit (Roche) and the Light Cycler 2.0 (Roche) were employed to perform the amplifications of *MGAT4A *and β-actin target, according to manufacturer's instructions. The Light Cycler software version 4.0 was used to analyze the fluorescence and number of cycles of each reaction. One point *MGAT4A *mRNA concentration was made in white cells from T2D and T2NB subjects. The transcript concentration was the average of triplicate determinations and was expressed as a normalized value to the β-actin mRNA levels for each individual.

### Determination of mRNA half-life

Detection of a 323 bp *MGAT4A *and a 587 bp β-actin RT-PCR products in the same cells were used to estimate mRNA half-lives in Jurkat T cell line. The cell cultures were treated with 1 μM Actinomycin D (Sigma-Aldrich, USA) to inhibit the transcription. Twenty four hours before treatment the cells were transferred from the 75 mL tissue bottle to 10 cm culture dishes in aliquots of 2.2 × 10^6 ^cells. Under these conditions we performed three independent experiments where cells were collected every 2 h and total RNA was isolated as described before.

### Statistical Analysis

The anthropometric and biochemical measurements are expressed as means ± SD, whereas gene expression measurements are expressed as means ± SE. The analysis of the data were made by ANOVA and tested for differences between groups using the Kruskal-Wallis analysis. *P *≤ values 0.05 were considered to be significant. Statistical operations were performed using the Stat View v4.57 (SAS Institute Inc., USA) and Prism 4 v4.02 (GraphPad Software, USA).

## Authors' contributions

ELO performed all the experiments of the study. MC was responsible for the patients' clinical data base, Genomic DNA database, data analysis and manuscript preparation. JGM was responsible of the general experimental design, data analysis and manuscript preparation. All authors read and approved the final manuscript.
